# Evaluating Multimedia and Language Tasks

**DOI:** 10.3389/frai.2020.00032

**Published:** 2020-05-05

**Authors:** Ian Soboroff, George Awad, Asad Butt, Keith Curtis

**Affiliations:** ^1^Retrieval Group, Information Access Division, Information Technology Laboratory, National Institute of Standards and Technology, Gaithersburg, MD, United States; ^2^Department of Physics, Georgetown University, Washington, DC, United States; ^3^Department of Computer Science, Johns Hopkins University, Baltimore, MD, United States

**Keywords:** evaluation, multimedia, information retrieval (IR), annotation, metrics

## Abstract

Evaluating information access tasks, including textual and multimedia search, question answering, and understanding has been the core mission of NIST's Retrieval Group since 1989. The TRECVID Evaluations of Multimedia Access began in 2001 with a goal of driving content-based search technology for multimedia just as its progenitor, the Text Retrieval Conference (TREC) did for text and web^1^.

## 1. Introduction

The recent article, “Challenges and Prospects in Vision and Language Research” by Kafle et al. (2019) identified several deficiencies in existing research in multimedia understanding. Existing benchmark tasks exhibit bias, are not robust, and induce spurious correlations which detract from rather than reveal advances in vision and language algorithms. These tasks frequently conflate a number of component tasks, such as object identification and entity coreference, which should be evaluated separately. Existing metrics such as BLEU and ROUGE used in automatic video captioning, question answering, and other tasks are not appropriate for these tasks.

We are currently experiencing a surge in the research and development of algorithms for visual and linguistic understanding. This surge was kick-started by the development of efficient training strategies for deep neural architectures, and the early successes of those architectures on existing benchmark datasets. But just as in open-source software where “given enough eyeballs, all bugs are shallow,”^2^ rapid hill-climbing on existing data can make the shortcomings in that data very clear.

The issues observed by Kafle et al. are not new; data-driven research communities in information retrieval and natural language processing have been sounding similar alarms for years (Allan et al., 2003; Radev et al., 2003; Nenkova and Passonneau, 2004; Gillard et al., 2006; Jones, 2007; Voorhees, 2008; Nenkova and McKeown, 2011; Carterette, 2015; Lommel, 2016; Culpepper et al., 2018; Ferro et al., 2018, to name a very few in the recent past). Frustration with available data, existing metrics, and accepted methodologies in evaluating artificial intelligence tasks seems as old as the field itself. However, in contrast with Kafle et al., we do not think aiming for a “visual Turing Test” is a solution. Turing Tests and their variants are themselves beset with evaluation difficulties that begin with asking “What is intelligence anyway?” and don't stop. Thus far, no test has been proposed that can distinguish truly intelligent understanding from an algorithm suitably advanced enough to be worth testing.

Rather, our group at NIST has found that embedding technology researchers within the process of developing the datasets, metrics, and methods used to evaluate that technology can create a cycle wherein the technology advances along with our understanding of the capabilities of that technology, how people might use it to improve their everyday lives, and how we would know if that were true.

The TRECVID evaluations of video access^3^ are an annual international evaluation activity to encourage research in video information retrieval. TRECVID provides datasets, uniform scoring procedures, and a forum for organizations to compare their results. The datasets are produced as an outcome of the evaluation process itself. By linking the research in visual understanding to the development of methods for measuring the degree of that understanding, we can continually improve our datasets and tasks.

## 2. Background

In 1991, DARPA approached NIST with the task of designing a search dataset with half a million documents, totalling 2GB of text. Don't laugh—in 1991 it cost around $10,000 for a big enough disk to hold 2GB of text and the additional data structures needed for searching^4^.

Search datasets, also called test collections, consist of a set of documents to search, a set of queries or information needs, and a list of the right documents to retrieve for each query. In our everyday web world we are typically only interested in the top one or two hits, but the kind of searchers people were interested in back then were *recall-oriented*: they might have tens or hundreds of relevant documents out of a set of millions, and they wanted to be able to find them all.

At that time, no research was happening at that scale. Commercial search technology was focused on Boolean queries over metadata and abstracts in patents, law, and (to some degree) financial news, with a few key global players. The research world was trying to invent something different: full text search, natural language queries, and results ranked in order of relevance. Research test collections were small, under 10,000 documents typically, and there were only a few of them, because no one was paying to build them and so universities were hand-labeling data which they then jealously guarded. Because they were interested in recall, it was felt that all the documents should be labeled for relevance against each query (Cleverdon, 1991), and that simply can't scale past tens of thousands of documents.

NIST proposed to only label the top-ranked results of the systems being evaluated, based on research proposals that suggested this “pooling” approach could find the vast majority of relevant documents, and the remainder could be assumed to be irrelevant. NIST also proposed that, instead of having a traditional closed DARPA evaluation, that research teams from all over the world be invited to participate openly. This was critical, because for pooling to work there needs to be a wide range of systems reflecting the range of the state of the art represented in the pools. DARPA agreed, and the first Text Retrieval Conference (TREC)^5^ was held in 1992 (Voorhees and Harman, 2005).

Initially, the evaluation tasks were “*ad-hoc* retrieval,” what we would today call search ranking, and “routing,” a task where queries were fixed and trained with some number of labels, and the remaining documents were routed to relevant queries. In 1996, TREC initiated a number of “tracks,” including filtering, interactive search, web search, and more, reflecting the growing interest in different tasks and creating datasets for them. In 2000, a video track started, and this track spun out in 2003 to become its own venue, TRECVID (Voorhees et al., 2014).

Data supporting all of these efforts was collected by NIST and labeled in the context of each task by contractor staff working under NIST technical supervision and using annotation tools designed specially for each task. We have found that investing in qualifications, training, and tools saves us time and expense in data cleaning, spam detection, and label verification.

## 3. TRECVID

Once it became it's own separate venue in 2003, TRECVID began with four tasks, each focused on some facet of the multimedia retrieval problem: shot boundary determination, story segmentation, high-level feature extraction, and search. The range of tasks was a deliberate move to bring together problems that were felt to be “low-level” or fundamental, component technologies alongside “high-level” problems that were directly motivated by an actual end-user task. Other tasks on this spectrum have included semantic indexing (high-level feature detection) and instance search (finding known items such as a person or location).

The TRECVID workshop has been held every year and typically hosts between four and six different tasks. These tasks support the multimedia research community by creating the infrastructure such as test collections necessary for task-specific research. The current slate of tasks include:

**Adhoc search:** As is common in text, retrieving relevant videos given a textual, still image, or video query;

**Instance search:** Searching for people, locations, and objects within a “closed world”;

**Video-To-Text:** Descriptive caption generation for short Internet videos;

**Activities in Extended Video:** Searching for complex activities that span multiple shots;

**Video Summarization:** Automatically creating short videos to summarize longer videos;

**Disaster Scene Description and Indexing:** Bringing concept detection and captioning to the domain of emergency management and disaster recovery.

### 3.1. Task History

From 2003 through 2006 TRECVID supported experiments in automatic segmentation, indexing, and content-based retrieval of digital video using broadcast news in English, Arabic, and Chinese. TRECVID also completed two years of pilot studies on exploitation of unedited video rushes provided by the BBC. From 2007 to 2009 TRECVID provided participants with cultural, news magazine, documentary, and education programming supplied by the Netherlands Institute for Sound and Vision^6^. Tasks using this video included segmentation, search, feature extraction, and copy detection. The BBC rushes were incorporated into a formal summarization task. Lastly, airport security video provided by the UK Home Office was used to support evaluation of event detection in surveillance video.

Up until 2010, TRECVID used test data from a small number of known professional sources—broadcast news organizations, TV program producers, and surveillance systems—that imposed limits on program style, content, production qualities, language, etc. In 2010 TRECVID confronted known-item search and semantic indexing systems with a new set of Internet videos characterized by a high degree of diversity in creator, content, style, production qualities, original collection device/encoding, language, and so forth. The videos are licensed under Creative Commons^7^ and were obtained from the Internet Archive^8^, giving the dataset its name, IACC. The videos have associated keywords and descriptions provided by the donor. The only selection criteria imposed by TRECVID beyond the Creative Commons licensing is one of video duration: they are short (<6 min). In addition to the IACC data set, NIST began developing an Internet multimedia test collection (HAVIC) with the Linguistic Data Consortium and used it in growing amounts (up to 8,000 h) in the TRECVID 2010–2017 Multimedia Event Detection (MED) task. The airport security video, introduced in TRECVID 2009, was reused each year until 2017 within the Surveillance Event Detection (SED) task.

In 2013, the BBC provided TRECVID with video programming from their long-running EastEnders^9^ series. EastEnders provides a “closed world” of people, locations, and objects, and this data is used for the instance Search (INS) task starting in 2013 and a new Video Summarization task that begins this year. The IACC collection was succeeded by an additional 600 h of Internet Archive video (IACC.2) which supported the Semantic Indexing task from 2013 to 2015 with new test data each year. In addition, a new concept localization (LOC) task was introduced in 2013 and continued up to 2016. In 2015 a new Video Hyperlinking task (LNK) previously run in MediaEval^10^ was added and then updated in 2018 to address social media storytelling linking.

From 2016 to 2018 the Adhoc Video Search (AVS) succeeded the Semantic Indexing task, with a new IACC.3 dataset (600 h) with maximum video duration of 9 min. A new pilot “Video to Text” (VTT) task was introduced in 2016 to address matching and describing videos using textual descriptions. Most recently, a new Creative Commons web video collection from Vimeo was released in 2019 to continue the Adhoc Video Search (AVS) task.

**Figure 1:**
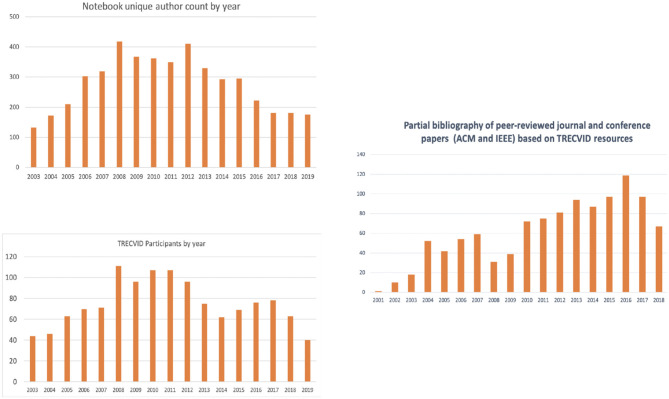
TRECVID teams, authors, and peer-reviewed academic publications by year.

Figure 1 show the history of TRECVID in terms of number of teams, unique author counts, and number of peer reviewed publications based on TRECVID provided resources from 2003 till 2019 (Thornley et al., 2011). The average number of teams participated across the years is 75 teams from academia and industry while number of authors from all teams ranged between about 100 and 400 team members in any year. Number of publications based on TRECVID resources also has been increasing year over year with the exception of previous couple of years due to the abundance of image and video data recently and the launch of several multimedia challenges.

### 3.2. Non-TRECVID Datasets

In this section we will review some of the most commonly used image and video datasets created outside TRECVID and used by researchers in different benchmarks as well as within TRECVID for the generic video search and instance search tasks.

One of the most well-known and heavily used datasets by the vision community is ImageNet (Deng et al., 2009). The image dataset is organized according to the WordNet hierarchy. Each meaningful concept in WordNet is called a “synonym set” or “synset.” There are more than 100,000 synsets in WordNet, and the majority of them are nouns (80,000+)^11^. In ImageNet, the aim is to provide on average 1,000 annotated images to illustrate each synset. ImageNet labels are crowdsourced from Amazon Mechanical Turk.

In the Oxford 5k dataset (Philbin et al., 2007), 5,062 images were collected from Flickr for particular Oxford landmarks. They were manually annotated to generate a comprehensive ground truth for 11 different landmarks, each represented by five possible queries. For each image and landmark, one of four possible labels was generated:

**Good** A nice, clear picture of the object/building.

**OK** More than 25% of the object is clearly visible.

**Bad** The object is not present.

**Junk** Less than 25% of the object is visible, or there are very high levels of occlusion or distortion.

In total, there are between 7 and 220 good and OK images per query. The Stanford Mobile Visual Search (SMVS) dataset (Chandrasekhar et al., 2011) consists of images for many different categories captured with a variety of camera-phones, and under widely varying lighting conditions. Database and query images alternate in each category, while the FlickrLogos-32 dataset^12^ contains photos showing brand logos and is meant for the evaluation of logo retrieval and multi-class logo detection/recognition systems on real-world images. The authors collected logos of 32 different logo brands by downloading them from Flickr where all logos have an approximately planar surface. The University of Kentucky retrieval benchmark (UKB) is a dataset (Nister and Stewenius, 2006) which consist of 2,550 classes, each class with four images in JPEG format. The pictures are from diverse categories such as animals, plants, and household objects.

One major difference between datasets from TRECVID and those from other benchmarks is that in TRECVID, it's usually the case that data collection happens first, followed by query development. This workflow makes the nature of the data very wild and different than other benchmarks' datasets which in most cases starts by defining queries first, and then collect the data. In the object detection and instance search tasks, TRECVID has focused on retrieving specific objects, persons, locations and their combinations, while other benchmarks have mainly focused on logos and landmarks. TRECVID mainly adopts video data while other benchmarks mainly use images. Due to the wild nature of the data in TRECVID, we see different scales in images and target query frequencies varies widely in the ground truth. In contrast, other benchmarks aim for a stable distribution of targets over queries, and similar scales where the target object is the main part of the image. Balanced categories are good for training and measuring classifiers, but in end-user applications the classes of interest are usually highly imbalanced.

We find similar differences with ImageNet in the domain of generic image/video search. ImageNet labels are exclusive (each image has a single label) and well-balanced across categories, while TRECVID labels and concepts are hierarchical, non-exclusive, and highly imbalanced. In ImageNet, the primary task is to find a label for an image, while TRECVID asks systems to find shots relevant to a label/concept. ImageNet targets the top-N error rate (For *N* = 1…5) while TRECVID measures the average precision of the full ranking, a recall-oriented measure. Finally while ImageNet provides typical examples, TRECVID examples are much less uniform.

Video captioning, or video-to-text, is a new task with growing popularity. In developing the TRECVID video-to-text task and datasets, we have attempted to overcome many of the problems that plague datasets in this area (Awad et al., 2019). A testing dataset of approximately 2000 videos is made available to the participating teams every year since 2016. The videos are annotated in-house by dedicated annotators. To the best of our knowledge, this is the only dataset for video captioning that uses dedicated annotators. In contrast, the other major datasets such as MSVD (Chen and Dolan, 2011) and MSR-VTT (Xu et al., 2016) use crowdsourcing to create their ground truth. An advantage of using dedicated annotators is that they receive in-person training and the task organizers have better oversight over them. The annotators are asked to include and combine into 1 sentence, if appropriate and available, four facets of the video they are describing:

**Who** is the video showing (e.g., concrete objects and beings, kinds of persons, animals, or things)?

**What** are the objects and beings doing (generic actions, conditions/state, or events)?

**Where** is the video taken (e.g., locale, site, place, geographic location, architectural)?

**When** is the video taken (e.g., time of day, season)?

This process results in captions that clearly explain the video, while no two ground truth captions are exactly the same. Datasets using crowdsourced ground truth captions usually end up with very generic captions, and frequently videos have multiple captions that are exactly the same. This can result in algorithms being trained on patterns that are specific to the dataset and do not generalize well. The comparison of the average sentence lengths for the major video-to-text datasets is as follows:

- MSVD: 7.03
- MSR-VTT: 9.28
- TRECVID-VTT: 19.09

**Figure 2:**
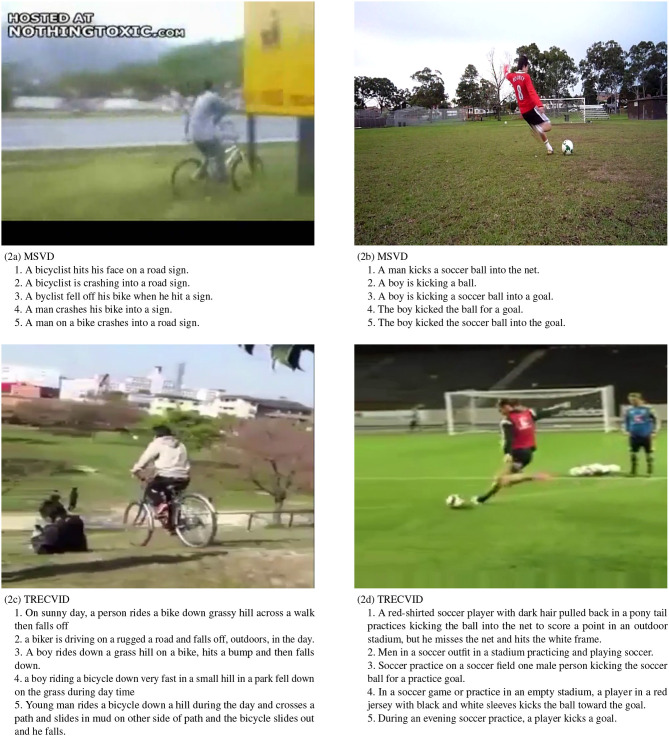
Comparison of video captions between the MSVD dataset and our TRECVID VTT dataset. The top row shows screenshots of two videos from the MSVD dataset, along with five captions. The bottom row shows screenshots of comparable videos from the TRECVID dataset and their corresponding captions.

This helps illustrate the contrast between the quality of descriptions. Figure 2 shows the comparison of video captions for sample videos from the MSVD dataset and the TRECVID dataset.

## 4. Automatic and Manual Evaluation

When building a dataset, deciding how to label it is a decision with overarching consequences. While it would be wonderful if data could be labeled completely automatically, that seems unlikely as long as we need the labeled data to improve the systems that would notionally provide the labels.

As alluded in section 2, the earliest datasets were labeled exhaustively and manually; see Cleverdon (1991) for historical perspective from the Cranfield Index Tests in the early 1960s. These experiments were evaluating the efficacy of computer-constructed library catalogs: were the terms in the documents better for search than hand-assigned “index terms”? It seems that the concern was exhaustivity—if there was a relevant document that was not labeled, then the indexing schemes would not be able to be measured when that document was desired.

Practically, this makes datasets of any compelling size impossible; to label the entire ClueWeb collection (733M web pages)^13^ for a single search query would take almost 700 years, assuming 30 s per web page. The pooling approach used in TREC is a reasonable alternative in many cases, and one that has been compared positively to statistical sampling (Aslam et al., 2006; Yilmaz and Aslam, 2008), static (Büttcher et al., 2007), and on-line machine learning (Abualsaud et al., 2018); in the context of different (Voorhees, 1998) and even erroneous labels (Carterette and Soboroff, 2010); and subject to complex pool selection criteria that seek to optimize the process (Voorhees, 2018).

Manual labeling is tractable and moderately scalable, if we only have to label data once. In cases where the system output is not a list of items in the collection, but rather a novel output, such as is in machine translation, snippet generation, summarization, captioning, and scene description, the system output itself must be labeled if we want to measure the quality of the output. Of course, the goal of evaluation is to improve the system output, and so we must review each new output of the system while taking care to avoid bias toward the new system.

There are two approaches to this review, labeling, and evaluation process: it can either be *extrinsic* or it can be *intrinsic*. For an extrinsic evaluation, the output is given to a person who must then accomplish some task with it, say produce a report or find more relevant information or identify key objects. If the person's task is easier to measure than the system output, perhaps because it can be compared to a gold-standard output, then extrinsic labeling is easier. Extrinsic evaluation also has the advantage of measuring the system in the context in which we expect it to be used, and so improvements in the system are directly measurable as improvements in the application. Of course, to do an extrinsic evaluation we need to have access to qualified people who use this application. Frequently in information technology we find ourselves working to invent new applications no one has imagined using before!

In an intrinsic evaluation, the output is assessed outside of the context of its use, purely on its own merits according to some model of correctness, relevance, or utility. For example, in machine translation, people are paid to create a number of reference translations, and then the system outputs are compared to the reference translations. In summarization, key concepts that a summary must contain can be manually identified in advance, and during evaluation we align summaries against the key concepts to measure coverage. The alignment or comparison step is again manual, but there has been a wealth of research proposing automatic methods, of which BLEU (Papineni et al., 2002), ROUGE (Lin, 2004), METEOR (Banerjee and Lavie, 2005), and other metrics are some of the most well-known. BLEU essentially measures the overlap of token *n*-grams between the output and several reference translations.

Automatic evaluation for generated natural language text has proven to be extremely difficult. Image and video captioning tasks often leverage BLEU and its cousins from machine translation. Metrics such as CIDEr (Vedantam et al., 2015) and SPICE (Anderson et al., 2016) have been created by the computer vision community to specifically solve the captioning problem. However, as noted by Kafle et al. (2019), these metrics often disagree with human evaluation, as well as among themselves. It is essential that better automatic metrics are developed to solve this problem.

While we attempt to find these better metrics, there is a large amount of research being done to improve captioning technology, and they use the existing metrics to evaluate their performance. Recognizing the problem, at TRECVID, we decided to include a manual evaluation, known as Direct Assessment (DA) (Graham et al., 2018) to selected submissions. The basic methodology is to present human assessors with a video and a single caption. The assessors then rate the caption on a scale of 1–100. Currently, this assessment is used in conjunction with the automatic metrics so they can be compared.

An alternative approach is to design tasks such that the output is in a simple to evaluate format. The visual question answering (VQA) is one such task where systems view an image or video and answer a natural language question (Antol et al., 2015). Since the target answer is only a few words, or from a closed set of answers, it is easier to evaluate the systems automatically (Antol et al., 2015). The TRECVID VTT task also includes a subtask where instead of generating captions, systems are provided a randomized list of human-generated captions, and asked to rank them for every video. This output is amenable to automatic evaluation, since we are only concerned with the ranks given to the correct captions. Hence, the two subtasks (matching and ranking, and description generation) can provide a clearer picture of the capability of competing systems.

In order to make usable automatic metrics, they need to be calibrated against manual assessments that reflect the quality of the result in the environment where it will be used. Machine translation started taking these steps when it was asked what BLEU score was required for a human to be able to understand the translation. Since measuring understanding is hard, it's better to see if the human can use the translation in such a way as it demonstrates understanding.

## 5. Designing Evaluation Tasks

In order to ensure that the metrics are actually targeting the real-world improvement you're working toward, the intrinsic evaluation needs to be aligned with an extrinsic evaluation, which can then be aligned with user studies and A/B tests in production systems. Clearly researchers aren't all expected to operate along that entire spectrum, but some groundwork needs to be laid if we wish to trust that improvements in the automatic metric actually leads to improvements in a real-world scenario.

One method for doing this is *task-driven evaluation*. Rather than starting from the system, we start from the person, and consider their task which we are trying to improve. If people currently perform that task, we can study those people, but if we are inventing new technology that supports an entirely new task, like content-based video search, we might have to mock it up with a model. It's essential that the task be something that a person actually does, with some goal in mind, and that the output be measurable in some way.

An example of a real-world task in information access is writing a report based on primary sources. This is a task undertaken by any number of kinds of knowledge workers, such as journalists, students, financial analysts, and doctors, every day. The form of the report clearly differs from domain to domain, and since the report is the measurable outcome, our task model is going to have to be at least somewhat domain-specific. Let's consider a financial analyst (person) that follows technology news and market data (primary sources) in order to make investment recommendations (report).

Given this task model, the next step is to create an abstraction of this task that we can experiment on. Measuring the quality of reports would seem to require a lot of manual review by financial experts, whose time is expensive, so perhaps we can make the task abstract by focusing on the collection of primary sources. Let's make that assumption explicit, along with assumptions that finding more correct primary sources results in a better report, missing relevant information makes the report worse, and wading through lots of junk makes the report worse, or at least makes the process of writing the report take longer.

It should be becoming clear that we are outlining an information retrieval task, but the essential thing to notice is that we are starting from the user, not the data or the system, and along the way, as we make assumptions and abstractions of a real-world activity, we do so explicitly, aligning the abstraction to decisions that will influence our choice of metrics.

We could take a different branch, and consider the process of writing the report as summarizing the primary source material. We could remove the variables of the quantity and quality of source material found by just providing the source material, already collected, to the system. Writing a report is a process of taking the information in the source material and producing an output that conveys the information without requiring that the reader know the primary sources. We could make some further abstractive assumptions, for example that the report will be just a sequence of distilled extractions from the source material, whenever a key financial datum or fact is mentioned, along with enough context to make it understandable. Leaving things out would result in a worse report, as would repeating information, or rendering facts incomprehensible by slicing up the initial sources poorly. As we get to an extractive summarization task, I have a task abstraction that stretches back to the actual user task, and I'm making assumptions that will guide us to a metric, but in the meantime they lay out a clear path back to our measurable concept of task success: a high-quality report.

In the context of content-based video search, following an intrinsic evaluation model, TRECVID always targeted real-world tasks that users wish to perform but technology is lacking. For example in the known-item search task, it simulates a user who remembers watching a video a while ago but can't find it in his video collection. An automatic system should (if exists) helps in retrieving this particular video given a textual description from the user of the part he/she remembers. On the other hand, the instance search task simulates a user who has an image or video example of something (object, person, location) and would like to find more instances or information of that specific thing. This particular scenario can be very useful in domains such as of law enforcement, library archives, logo, and brand protection. In the known-item search task, human assessors actually watched video clips and after few weeks were asked to write a description to simulate the real task, while in the instance search task a suitable dataset (BBC Soap Opera Eastenders series) with reasonable number of repeated instances was essential to simulate the task.

The choice of metrics is driven by the abstraction of the task. We can focus on those key items that need to be retained in an extractive summary, having people identify those key elements and then match them to the produced summary, which then leads us to overlap metrics like the Jaccard measure. This is the pyramid method for summary evaluation (Nenkova and Passonneau, 2004), which was compared to the ROUGE automatic metric by Owczarzak et al. (2012).

In contrast, achieving “intelligence” is unworkable as an evaluation task. Intelligence is notoriously hard to measure in humans, with well-documented biases for age, income, social position, health, and culture. While some areas of AI such as chatbots are dominated by trying to decide whether the output seems natural or could be confused with the output of a person, it isn't possible to measure how natural or how different the output is, beyond what can be done with tools like BLEU. Without a consistent measurement, it's not clear how to optimize the algorithms creating those outputs.

## 6. Conclusion

Evaluation-driven research, using datasets to measure and improve the quality and effectiveness of algorithms, has grown from the early days of computer science to dominate the development of artificial intelligence. Along the way, this process itself has become an important subject of study. Evaluation workshops, data challenges, and even leaderboard competitions can be forums for improving our datasets just as we improve our systems.

A critical part of making good datasets is grounding the evaluation task firmly in a user task, something a person does, and which we hope to improve through technology. This abstraction process involves a number of assumptions and abstractions, and whenever one assumption is made, some others are probably not mentioned but assumed as well. The open evaluation workshop is a social process for eliciting, identifying, exploring, and testing those assumptions. For datasets that come engraved on stone tablets from the top of a mountain, there is no such process.

In their paper, Kafle et al. (2019) make the argument that creating better datasets and evaluation techniques is crucial to the progress of vision and language tasks, and more research is needed in this area. We wholeheartedly agree with this conclusion, and in this response hope to have shared some methods for achieving that goal.

## Author Contributions

IS was approached to write the paper, structured it, and wrote sections Introduction, Background, Automatic and Manual Evaluation, and Designing Evaluation Tasks. GA wrote section TRECVID. AB wrote section Non-TRECVID Datasets and parts of section Automatic and Manual Evaluation. KC contributed to sections TRECVID and Automatic and Manual Evaluation.

## Conflict of Interest

The authors declare that the research was conducted in the absence of any commercial or financial relationships that could be construed as a potential conflict of interest.

## Footnotes

^2^en.wikipedia.org/wiki/Linus's_law

^3^trecvid.nist.gov

^4^Now it could be a browser demo. *Sic transit gloria mundi*.

^5^trec.nist.gov

^6^https://www.beeldengeluid.nl/en

^7^creativecommons.org/licenses/

^8^archive.org

^9^www.bbc.co.uk/programmes/b006m86d

^10^multimediaeval.org

^11^http://image-net.org/about-overview

^12^https://www.uni-augsburg.de/en/fakultaet/fai/informatik/prof/mmc/research/datensatze/flickrlogos/

^13^https://lemurproject.org/clueweb12/
